# Magnetic iron oxide nanoparticle-loaded hydrogels for photothermal therapy of cancer cells

**DOI:** 10.3389/fbioe.2023.1130523

**Published:** 2023-03-16

**Authors:** Yunfei Ji, Chunpu Wang

**Affiliations:** ^1^ Department of Critical Care Medicine, Chengde Central Hospital, Chengde, Hebei, China; ^2^ Department of Cardiothoracic Surgery, Chengde Central Hospital, Chengde, Hebei, China

**Keywords:** iron oxide nanoparticles, hydrogels, cancer therapy, photothermal therapy, light

## Abstract

**Introduction:** Non-invasive photothermal therapy (PTT) is a competitive treatment for solid tumors, while the efficacy is largely dependent on the effective retention of photothermal converters in tumor tissues.

**Methods:** Herein, the development of iron oxide (Fe_3_O_4_) nanoparticle-loaded alginate (ALG) hydrogel platform for PTT of colorectal cancer cells is reported. Fe_3_O_4_ nanoparticles synthesized *via* coprecipitation method after reaction of 30 min have a small size (61.3 nm) and more suitable surface potential, and can mediate PTT under near-infrared (NIR) laser irradiation. The premix of Fe_3_O_4_ nanoparticles and ALG hydrogel precursors can be gelatinized by Ca^2+^-mediated cross-linking to form this therapeutic hydrogel platform.

**Results:** The formed Fe_3_O_4_ nanoparticles can be effectively taken up by CT26 cancer cells and induce the death of CT26 cells *in vitro* under NIR laser irradiation because of their excellent photothermal property. In addition, Fe_3_O_4_ nanoparticle-loaded ALG hydrogels show negligible cytotoxicity at the studied concentration range, but can significantly kill cancer cells after PTT effect.

**Conclusion:** This ALG-based hydrogel platform provides a valuable reference for subsequent in vivo studies and other related studies on Fe_3_O_4_ nanoparticle-loaded hydrogels.

## 1 Introduction

Cancer has long been one of the most lethal diseases that threat human health (Navya et al., 2019; Ding et al., 2022; Jing et al., 2022). Although surgery, chemotherapy, and radiotherapy are the mainstays of cancer treatment in the past, their limitations such as low specificity and high risk of recurrence, have forced researchers to divert their attention beyond these traditional treatments to explore more effective therapy (Meng et al., 2020; Caballero et al., 2022). Photothermal therapy (PTT) is a very promising cancer treatment modality emerging in recent years (Zhang et al., 2020; Dong et al., 2021). Utilizing photothermal converters to capture and transform external light, the local heat generated during PTT can induce intracellular protein denaturation and apoptosis. Especially for tumor tissues with dense blood vessels and hindered heat dissipation, PTT is an extremely promising method for tumor ablation (Cristofolini et al., 2016). Gold nanoparticles, carbon-based nanomaterials, and some small-molecule dyes have been used as photothermal agents for cancer PTT (Liu et al., 2007; Bardhan et al., 2011; Espinosa et al., 2016). Unfortunately, the applications of most of these materials are limited due to their low retention and potential toxicity (Espinosa et al., 2016). Therefore, it is very important to explore safe and degradable photothermal agents. Biodegradable iron oxide (Fe_3_O_4_) nanoparticles have been approved by the Food and Drug Administration (FDA) as a magnetic resonance imaging (MRI) contrast agent. In addition, the strong absorption of Fe_3_O_4_ nanoparticles in the near-infrared (NIR) window can be utilized for PTT (Shubayev et al., 2009; Anselmo and Mitragotri, 2015).

In general, many nanoparticles after intravenous injection are readily taken up by macrophages in the blood circulation and cleared by the reticuloendothelial system, thus hindering the aggregation of nanoparticles in tumors (Li et al., 2014). Therefore, the reliability of the carriers is of great significance for the performance of the anti-tumor ability of the nano-formulations. Hydrogels are a burgeoning class of three-dimensional polymer networks (Griffin et al., 2015; Vegas et al., 2016; Puiggalí-Jou et al., 2021). As a drug delivery system, hydrogels can not only release drugs controllably to fully exert anticancer efficacy, but also obviously weaken systemic toxicity in the form of intravenous administration. In addition, such hydrogel-based therapeutic platforms can reduce the numbers of drug administrations while maintaining biosafety (Almawash et al., 2022). Currently, alginate (ALG)-based hydrogels have enabled great advances in biomedicine due to their non-immunogenicity, excellent biocompatibility, and mild gel-forming conditions. ALG is a natural linear anionic polymer that can crosslink with divalent cations to form hydrogels (Lee and Mooney, 2012; Liu et al., 2021). By mixing with ALG hydrogel precursors and forming hydrogels after injection, many hydrophilic drugs and nanoparticles can be easily loaded and aggregated in tumor tissues with a relatively longer residence time after administration to exert therapeutic effects (Kim and Martin, 2006; Goncalves et al., 2014). However, the use of ALG hydrogels for loading of Fe_3_O_4_ nanoparticles for effective cancer PTT has not been explored.

In this work, a Fe_3_O_4_ nanoparticle-loaded hydrogel (Fe_3_O_4_ hydrogel) was constructed for cancer PTT. Fe_3_O_4_ nanoparticles were synthesized by chemical synthesis and mixed with ALG hydrogel precursors to form a homogeneous injectable solution. The Fe_3_O_4_ hydrogel was then successfully prepared *in vitro* by mixing with Ca^2+^ solution at a concentration similar to that in biological tissues. After NIR irradiation, the cell viability of CT26 cells co-incubated with Fe_3_O_4_ hydrogels was significantly lower than that in the control group, and the photothermal killing ability of the Fe_3_O_4_ hydrogels was not shielded by the ALG hydrogel carrier. The Fe_3_O_4_ nanoparticle-loaded hydrogels reported in this study significantly inhibited the viability of colorectal cancer cells.

## 2 Materials and methods

### 2.1 Materials

Ferrous chloride tetrahydrate (FeCl_2_ 4H_2_O), ferric chloride hexahydrate (FeCl_3_ 6H_2_O), sodium hydroxide (NaOH) and ALG were purchased from Shanghai Sinopharm Chemical Reagent Co., RPMI 1640 cell culture medium, fetal bovine serum (FBS), and penicillin-streptomycin were obtained from Gibco (Grand Island, NY, United States). Cell counting kit-8 (CCK-8) was purchased from Dojindo Laboratories (Kumamoto, Japan). Ultrapure water used in the experiments was prepared using a water purification system (PALL Cascada, MI, United States).

### 2.2 Characterization techniques

The UV-visible spectra of Fe_3_O_4_ nanoparticles were characterized by Persee spectrophotometer (TU-1810, Beijing, China). The surface morphologies of Fe_3_O_4_ hydrogels were observed using a scanning electron microscope (SEM, HITACHI, Japan). The hydrodynamic diameters and zeta potentials of Fe_3_O_4_ nanoparticles were measured using a Zetasizer Nano-series (Nano-ZS90, Malvern, United Kingdom). The Fe concentrations were measured by using an inductively coupled plasma atomic emission spectroscopy (ICP-AES) system (Hudson, NH, United States).

### 2.3 Synthesis of Fe_3_O_4_ nanoparticles

Fe_3_O_4_ nanoparticles were synthesized according to a previous report (Hu et al., 2015). In brief, 178 mg FeCl_2_ 4H_2_O and 314 mg FeCl_3_ 6H_2_O were dissolved in deionized (DI) water. Then 10 mL of NaOH solution (200 mg/mL) was added to the above solution. After mixing well, the solution was placed in a water bath at 80°C for 30 min or 2 h, respectively. The obtained samples were used to evaluate the influence of the reaction times on the properties of the nanoparticles. Subsequently, the mixed solution was placed on a magnetic stirrer to precipitate the synthesized Fe_3_O_4_ nanoparticles, and the upper liquid was discarded. Then Fe_3_O_4_ nanoparticles were dispersed in 10 mL water under sonication. The above steps were repeated at least 5 times to purify the Fe_3_O_4_ nanoparticles.

### 2.4 Synthesis of Fe_3_O_4_ hydrogels

To prepare Fe_3_O_4_ hydrogels, Fe_3_O_4_ nanoparticles were mixed with ALG solution to obtain AF mixture solution and the solution was then injected into Ca^2+^ (1.8 mM) solution. AF solutions at different concentrations of ALG (0.5, 1, 2.5, 5, 10 μg/mL) were slowly injected into the Ca^2+^ solution (1.8 mM). Then photographs were taken at different times after injection of solutions.

### 2.5 Evaluation of *in vitro* photothermal effect

To evaluate the photothermal properties of Fe_3_O_4_ nanoparticles and Fe_3_O_4_ hydrogels, 200 μL of Fe_3_O_4_ solution or Fe_3_O_4_ hydrogels at the Fe concentration of 200 μg/mL were placed in a 96-well plate. Then, 808 nm laser at different power densities (0.5, 1.0, and 1.5 W/cm^2^) was used to irradiate the solutions for 5 min. Thermal images of solutions were obtained using a thermal infrared camera and the temperatures of the solution were recorded under laser irradiation. To investigate the effect of Fe concentrations on the photothermal properties, Fe_3_O_4_ nanoparticles or Fe_3_O_4_ hydrogels at different Fe concentrations (100, 200, 350, 500 μg/mL) were irradiated by 808 nm laser (1 W/cm^2^) for 5 min. DI water was used as the control group. These solutions were irradiated by a laser at the power density of 1 W/cm^2^. The laser was turned on/off every 5 min for 50 min to evaluate the photothermal stability of Fe_3_O_4_ nanoparticles and Fe_3_O_4_ hydrogels.

### 2.6 Evaluation of *in vitro* cellular uptake

CT26 cancer cells were cultured in RPMI 1640 cell medium containing penicillin, streptomycin and 10% FBS at 37°C and 5% CO_2_. The cells were incubated with Fe_3_O_4_ nanoparticles at the Fe concentration of 100 μg/mL for different time. The cellular uptake efficacy was evaluated using ICP-AES.

### 2.7 Evaluation of *in vitro* cytotoxicity

The cytotoxicity of CT26 cancer cells after incubation with Fe_3_O_4_ nanoparticles or Fe_3_O_4_ hydrogels was investigated using the CCK-8 assay. CT26 cancer cells were cultured with 100 μL of fresh cell culture medium in 96-well plates (10,000 cells per well) and incubated for 24 h. The cell culture medium was then discarded and Fe_3_O_4_ solutions or Fe_3_O_4_ hydrogels (1 μg/mL for ALG) at different Fe concentrations (25, 50, 100, 200 and 400 μg/mL) were added into the cell culture medium. Meanwhile, 1 μL of Ca^2+^ solution (180 mM) was added into the wells containing Fe_3_O_4_ nanoparticles and ALG to form Fe_3_O_4_ hydrogels. After the incubation of cells for 24 h, the cell culture medium was discarded and the cells were carefully washed with PBS to remove free Fe_3_O_4_ nanoparticles. Cell culture medium containing 10% CCK-8 agent was then added into each well. After incubation of the cells for 2 h, the absorbance value of each well at 450 nm was detected using a microplate reader. The cells treated with PBS were used as a control. The ratio of absorbance values was used to calculate cell viability.

### 2.8 Evaluation of *in vitro* therapeutic efficacy

To evaluate the therapeutic effect of Fe_3_O_4_ nanoparticles and Fe_3_O_4_ hydrogels, CT26 cancer cells were seeded in 96-well plates (10,000 cells per well) and incubated at 37°C and 5% CO_2_ for 24 h. For Fe_3_O_4_ nanoparticle treatment group, the cell culture medium was discarded and 10 μL Fe_3_O_4_ nanoparticles at the Fe concentration of 200 μg/mL was added into each well containing 190 μL cell culture medium. For Fe_3_O_4_ hydrogel treatment group, the cell culture medium was discarded, and 10 μL solution of Fe_3_O_4_ nanoparticles (200 μg/mL) and ALG (the concentration of ALG was 1 μg/mL) was added into each well containing 189 μL cell culture medium, and then 1 μL of Ca^2+^ solution (180 mM) was added into the wells to form Fe_3_O_4_ hydrogels. The formed hydrogels could stick to cells for cell incubation. After incubation of cells for 12 h, the cells were irradiated by 808 nm laser (1 W/cm^2^) for 5 min. After that, the cells were incubated for another 12 h and the hydrogels were removed, and then cell viability was detected by CCK-8 assay.

### 2.9 Statistical analysis

Significant difference between the experimental statistics is analyzed by One-way ANOVA and indicated as (*), *p* < 0.01 by (**) and *p* < 0.001 by (***).

## 3 Results and discussion

### 3.1 Synthesis and characterization of Fe_3_O_4_ nanoparticles

**FIGURE 1:**
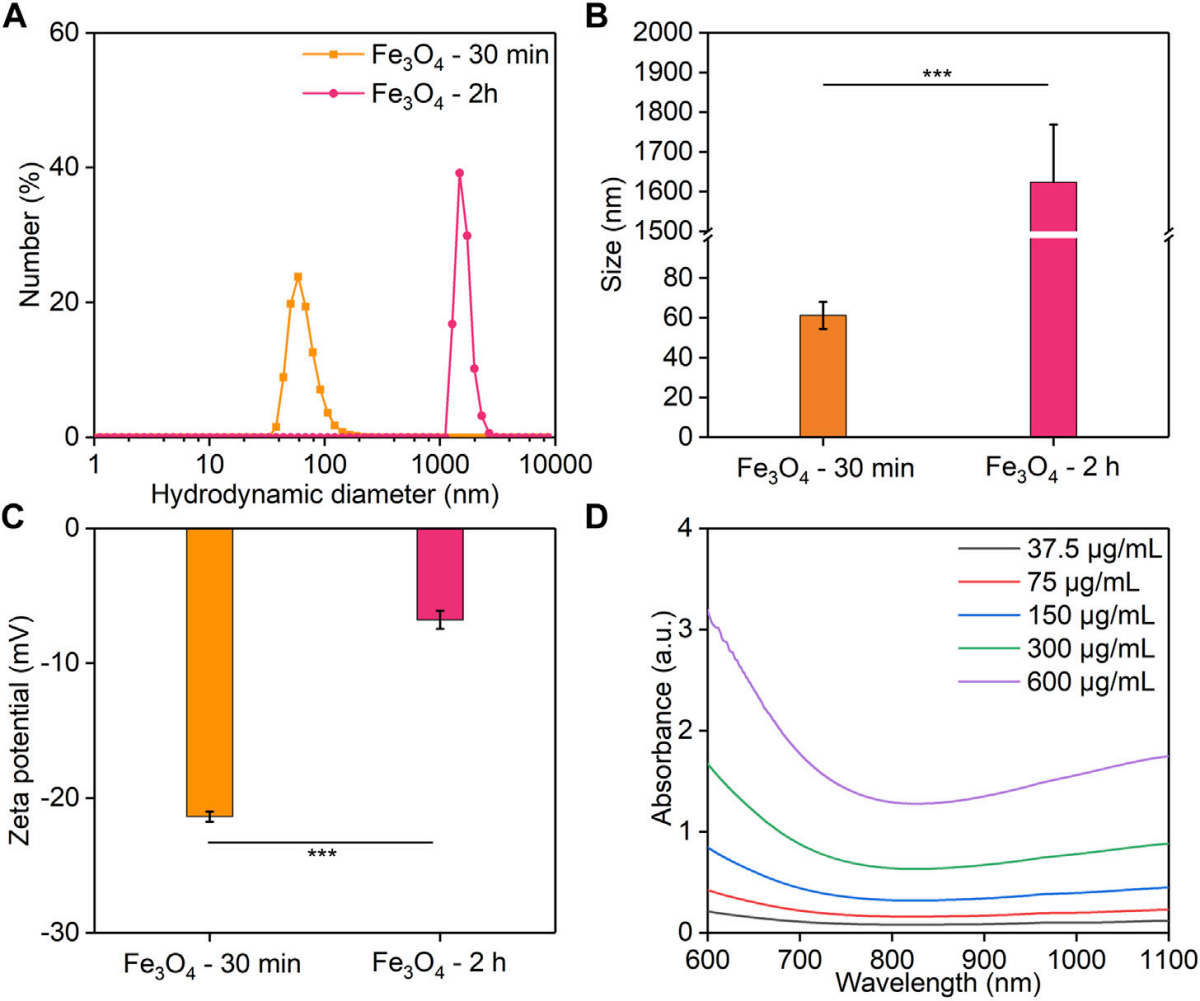
Characterization of Fe_3_O_4_ nanoparticles. **(A,B)** Hydrodynamic diameter. **(C)** The zeta potential of Fe_3_O_4_ nanoparticles obtained for a reaction time of 30 min or 2 h. **(D)** UV-visible spectra of Fe_3_O_4_ nanoparticles with different Fe concentrations.

To investigate the effect of different reaction times on the properties of Fe_3_O_4_ nanoparticles, the hydrodynamic sizes and surface zeta potentials of the Fe_3_O_4_ nanoparticles formed after the reaction for 30 min or 2 h were measured. The hydrodynamic diameter of Fe_3_O_4_ nanoparticles with 30 min of reaction was 61.3 nm, which was much smaller than that of 2 h (1,624 nm) (Figures 1A, B). Meanwhile, the surface zeta potential of Fe_3_O_4_ nanoparticles formed *via* a 30 min reaction (−21.4 mV) was lower than that of 2 h (−6.8 mV) (Figure 1C). These results indicated that the Fe_3_O_4_ nanoparticles obtained by reacting for 30 min had a smaller diameter and a more suitable surface potential. The Fe_3_O_4_ nanoparticles formed *via* a 30 min of reaction had a smaller size, and thus they would show a higher stability. Stronger steric stabilization and less electrostatic stabilization may lead to their lower surface potential (Shah et al., 2014). Therefore, the reaction time was set at 30 min in the following study. In addition, the UV-Vis absorption spectra of Fe_3_O_4_ nanoparticles were evaluated (Figure 1D). The absorbance value at 850 nm increased with increasing Fe concentrations measured using ICP-AES, which could enable their PTT applications (Yang et al., 2017).

### 3.2 Evaluation of the photothermal conversion efficacy of Fe_3_O_4_ nanoparticles

**FIGURE 2:**
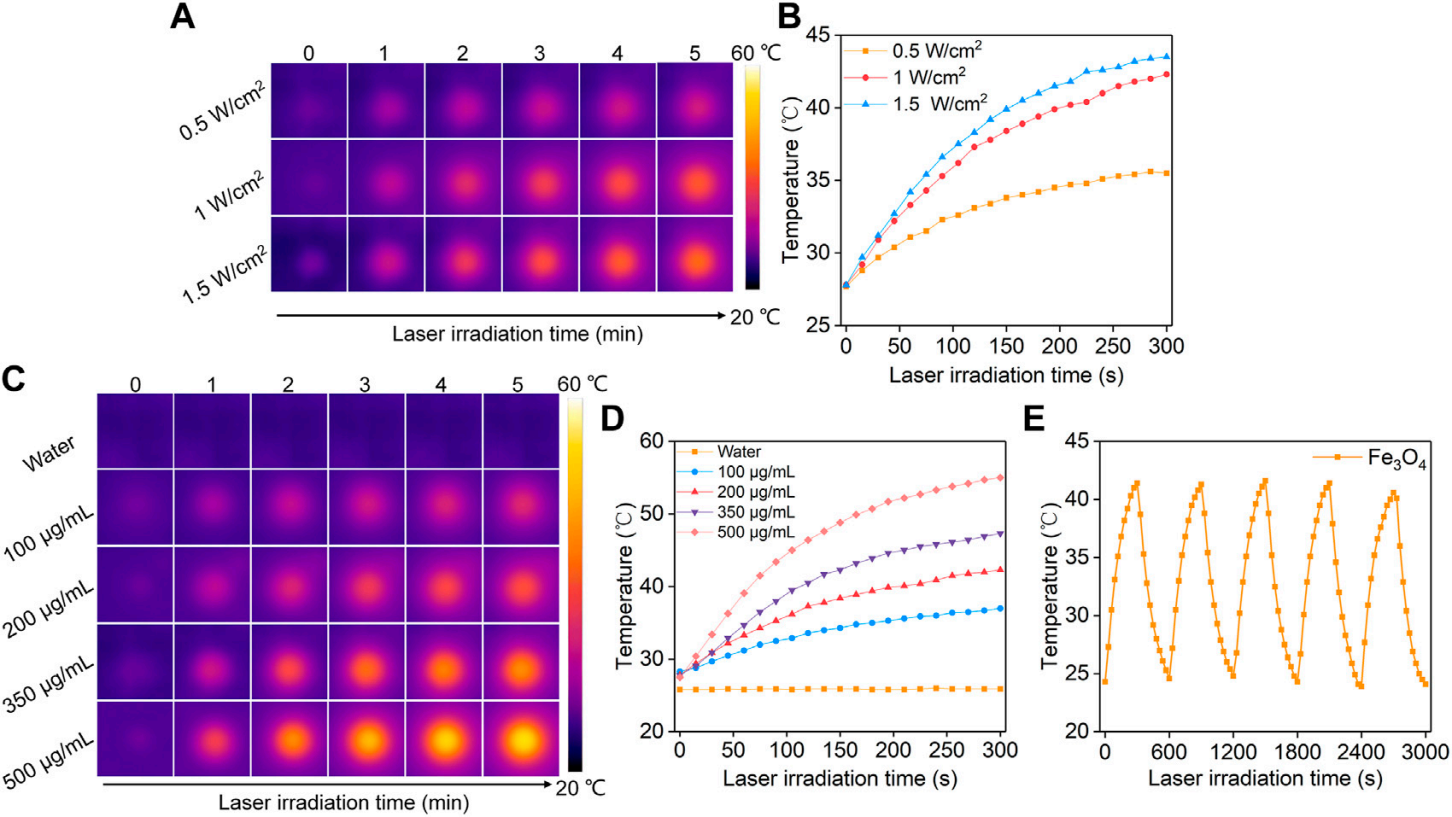
Evaluation of photothermal conversion efficiency. **(A)** Thermal imaging of Fe_3_O_4_ nanoparticle solution under 808 nm laser irradiation at power densities of 0.5, 1.0, and 1.5 W/cm^2^. **(B)** Temperature profiles of Fe_3_O_4_ nanoparticle solution under different power densities of 808 nm laser irradiation. **(C)** Thermal imaging of Fe_3_O_4_ nanoparticle solutions at concentrations of 100, 200, 350, and 500 μg/mL under 808 nm laser irradiation at a power density of 1.0 W/cm^2^. **(D)** Temperature profiles of Fe_3_O_4_ nanoparticle solutions at different concentrations under 808 nm laser (1.0 W/cm^2^) irradiation. **(E)** The photothermal stability evaluation of the Fe_3_O_4_ nanoparticle solutions after 5 laser cycles.

To evaluate the photothermal conversion efficacy, the Fe_3_O_4_ nanoparticle solutions were irradiated by an 808 nm laser. The thermal images were captured and the temperatures of the solutions were recorded. At the same Fe concentration, the temperatures of the solutions gradually increased with increasing laser time, which reached a maximum after 5 min of laser irradiation (Figure 2A). In order to evaluate the relationship between different power densities and the temperature increases, lasers at different power densities (0.5, 1, 1.5 W/cm^2^) were used. Higher power densities achieved a greater increase in solution temperatures. The solution temperature increased to 35.5, 42.3, and 43.5°C after 5 min of laser irradiation at the power densities of 0.5, 1, and 1.5 W/cm^2^, respectively (Figure 2B). The solutions at different Fe concentrations showed different degrees of temperature increases after irradiation by 808 nm laser (1 W/cm^2^) for the same time (Figure 2C). The temperature of solutions at Fe concentrations of 100, 200, 350, and 500 μg/mL increased to 37.0, 42.3, 47.3, 55.0°C, respectively (Figure 2D). In contrast, the temperature of PBS solution showed no significant change after laser irradiation. The photothermal stability of Fe_3_O_4_ nanoparticles was then evaluated. After five cycles of heating and natural cooling, the temperature increases of the Fe_3_O_4_ nanoparticle solutions did not change significantly, indicating their good photothermal stability.

### 3.3 Preparation and characterization of Fe_3_O_4_ hydrogels

**FIGURE 3:**
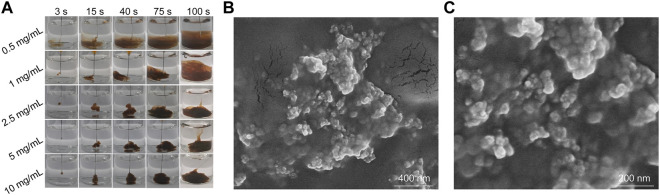
Characterization of Fe_3_O_4_ hydrogels. **(A)** Photographs of AF solutions at different ALG concentrations injected into Ca^2+^ solutions at different times. **(B,C)** SEM images of Fe_3_O_4_ hydrogels.

To prepare Fe_3_O_4_ hydrogels, Fe_3_O_4_ nanoparticles were added to ALG solutions at different concentrations (0.5, 1, 2.5, 5, 10 mg/mL) and the solutions were slowly injected into 10 mL Ca^2+^ solution (1.8 mM). The hydrogels could be formed *via* cross-linking of ALG by Ca^2+^. The rate of hydrogel formation increased with the increasing of ALG concentrations (Figure 3A). When the solution with a ALG concentration of 0.5, 1 or 2.5 mg/mL was injected into the Ca^2+^ solution, hydrogels could be formed. However, the formed hydrogels disintegrated rapidly in solution due to the low cross-linkage of the formed hydrogels. When the concentration of ALG was 5 or 10 mg/mL, the formed hydrogels were able to maintain stability state for a long time without significant morphological changes due to the high cross-linking degree. Therefore, the concentration of ALG was set at 5 mg/mL in the following experiments. The SEM images showed that nanoparticles were attached to the surface of the hydrogels, which proved that Fe_3_O_4_ nanoparticles could be effectively encapsulated into the hydrogels (Figures 3B, C).

### 3.4 Evaluation of photothermal conversion efficacy of Fe_3_O_4_ hydrogels

**FIGURE 4:**
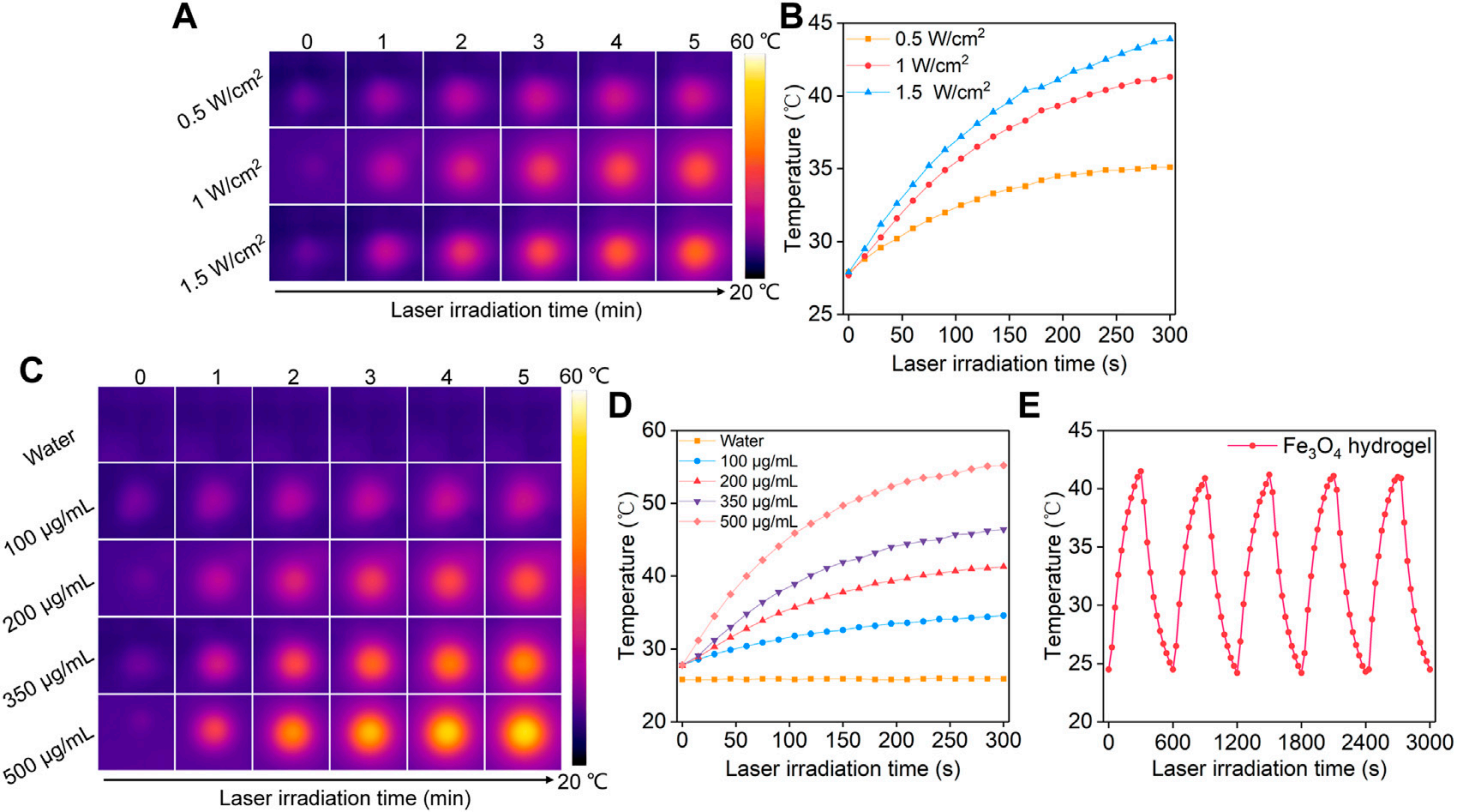
Evaluation of photothermal conversion efficiency of Fe_3_O_4_ hydrogels. **(A)** Thermal imaging of Fe_3_O_4_ hydrogels under 808 nm laser irradiation at power densities of 0.5, 1.0, and 1.5 W/cm^2^. **(B)** Temperature profiles of Fe_3_O_4_ hydrogels under 808 nm laser irradiation at different power densities. **(C)** Thermal imaging of hydrogels at concentrations of 100, 200, 350, and 500 μg/mL under 808 nm laser irradiation at a power density of 1.0 W/cm^2^. **(D)** Temperature profiles of Fe_3_O_4_ hydrogels at different concentrations under 808 nm laser (1.0 W/cm^2^) irradiation. **(E)** The photothermal stability evaluation of Fe_3_O_4_ hydrogels after 5 laser cycles.

The Fe_3_O_4_ hydrogels were irradiated using an 808 nm laser to study their photothermal conversion properties. The thermal images were captured and temperatures of the hydrogels were recorded. The temperatures of Fe_3_O_4_ hydrogel solutions gradually increased with the increasing of laser irradiation time, which reached the maximum after laser irradiation for 5 min (Figure 4A). Meanwhile, the temperatures of the hydrogel solutions irradiated by 808 nm laser at different power densities (0.5, 1, 1.5 W/cm^2^) for 5 min were different. The solution temperature increased to 35.1, 41.3, and 43.9°C after 5 min of laser irradiation at power densities of 0.5, 1, and 1.5 W/cm^2^, respectively (Figure 4B), indicating that higher power densities could achieve better photothermal effects. The temperature increases of the Fe_3_O_4_ hydrogel solutions were not significantly different for power density of 1 and 1.5 W/cm^2^, so the power density used in the subsequent experiments was set at 1 W/cm^2^. The photothermal performance of Fe_3_O_4_ hydrogels at different Fe concentrations was also evaluated. After 5 min of 808 nm (1 W/cm^2^) laser irradiation, the temperature of the hydrogel solutions at Fe concentrations of 100, 200, 350, and 500 μg/mL increased to 34.6, 41.3, 46.4, and 55.2°C, respectively (Figures 4C, D). These concentrations were used for photothermal effect evaluation because the Fe_3_O_4_ nanoparticles at these concentration ranges could obviously increase temperatures under laser irradiation (Chen et al., 2023). Meanwhile, the photothermal stability of the Fe_3_O_4_ hydrogels was evaluated (Figure 4E). The temperature increase did not change significantly after five cycles of heating/cooling, indicating that the Fe_3_O_4_ hydrogels had good photothermal stability. There was no significant difference between the photothermal performance of Fe_3_O_4_ hydrogels and Fe_3_O_4_ nanoparticles, indicating that the loading of Fe_3_O_4_ nanoparticles into hydrogels did not affect their photothermal performance.

### 3.5 Evaluation of *in vitro* treatment efficiency

**FIGURE 5:**
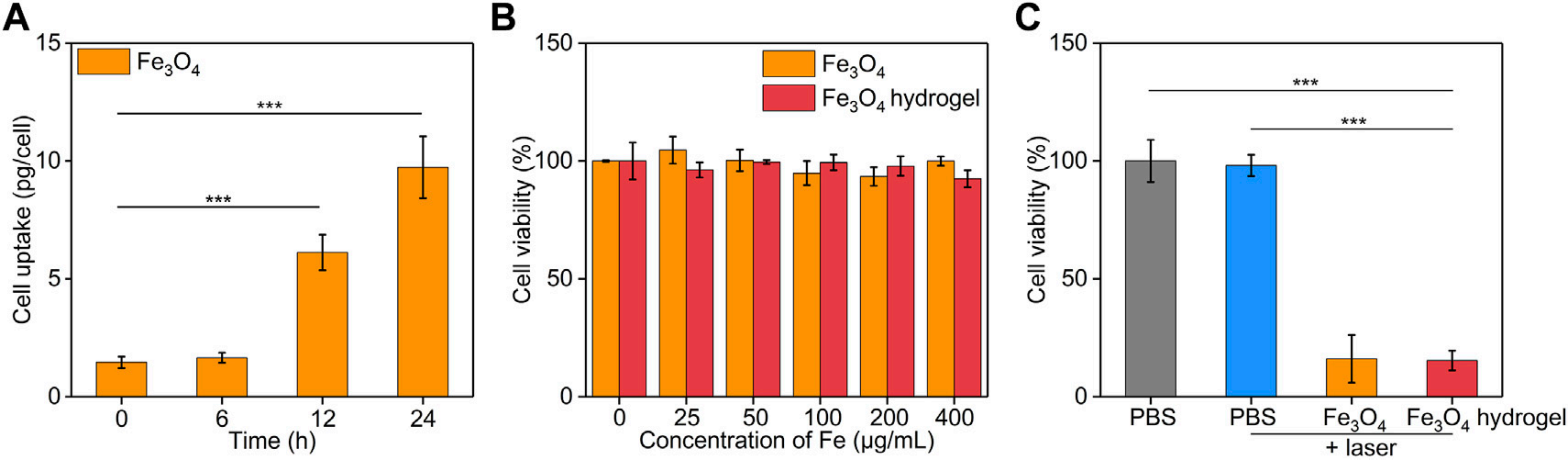
*In vitro* treatment efficacy evaluation. **(A)** Cellular uptake assay of Fe_3_O_4_ nanoparticles by CT26 cancer cells. **(B)** Cell viability of CT26 cancer cells after treatment with Fe_3_O_4_ nanoparticles or Fe_3_O_4_ hydrogels at different Fe concentrations for 24 h. **(C)** Cell viability of CT26 cancer cells after incubation with PBS, Fe_3_O_4_ nanoparticles, or Fe_3_O_4_ hydrogels with or without 808 nm laser irradiation (1.0 W/cm^2^, 5 min).

The *in vitro* cellular uptake of Fe_3_O_4_ nanoparticles by cancer cells was evaluated using ICP-AES. The cellular uptake of Fe_3_O_4_ nanoparticles was pivotal to induce therapeutic effect for Fe_3_O_4_ nanoparticle-treated cells. After incubation the cells with Fe_3_O_4_ nanoparticles, the Fe uptake in cancer cells gradually increased in a time dependent manner (Figure 5A). After 24 h, the cellular Fe level increased by 6.6-fold. These results suggested the effective cellular uptake of Fe_3_O_4_ nanoparticles by cancer cells. To evaluate the cytotoxicity, CT26 cancer cells were co-incubated with Fe_3_O_4_ nanoparticles or Fe_3_O_4_ hydrogels for 24 h. The cell viability of CT26 cells was higher than 92.5% after incubation with Fe_3_O_4_ nanoparticles or Fe_3_O_4_ hydrogels even when the Fe concentration was as high as 400 μg/mL (Figure 5B), indicating that both Fe_3_O_4_ nanoparticles and Fe_3_O_4_ hydrogels had good biosafety and cytocompatibility. The *in vitro* therapeutic effects of Fe_3_O_4_ nanoparticles and Fe_3_O_4_ hydrogels were then evaluated using CCK-8 assay. CT26 cancer cells were irradiated by 808 nm laser (1 W/cm^2^) for 5 min, and the cell viability was not significantly reduced compared to that in the control group, indicating that cancer cells were not killed by laser irradiation alone (Figure 5C). When CT26 cells were treated with Fe_3_O_4_ nanoparticles or Fe_3_O_4_ hydrogels plus laser irradiation, the cell activity of cells decreased to 16.1% and 15.4%, respectively. The cell vitality of cells in Fe_3_O_4_ nanoparticles + laser and Fe_3_O_4_ hydrogels + laser was similar. These results verified the therapeutic effect of Fe_3_O_4_ hydrogels. Although the therapeutic efficacy of Fe_3_O_4_ hydrogels was similar to that of Fe_3_O_4_ nanoparticles, the Fe_3_O_4_ hydrogels could maintain a high concentration at injected sites and obviously reduce systemic toxicity in the form of intravenous administration, which would contribute to their future *in vivo* studies.

## 4 Conclusion

Herein, we report the development of Fe_3_O_4_ nanoparticle-loaded hydrogel platform (Fe_3_O_4_ hydrogels) for PTT of colon cancer cells. The synthesis of Fe_3_O_4_ nanoparticles could be achieved after 30 min of reaction, and the formed Fe_3_O_4_ nanoparticles showed a smaller diameter, a more suitable surface potential and a good photothermal conversion efficacy under 808 nm laser irradiation. The cross-linking of ALG solutions containing Fe_3_O_4_ nanoparticles by Ca^2+^ led to the formation of Fe_3_O_4_ hydrogels. The obtained Fe_3_O_4_ hydrogels also showed a high photothermal conversion efficiency under 808 nm laser irradiation. Both Fe_3_O_4_ nanoparticles and Fe_3_O_4_ hydrogels were found to have good cytocompatibility. *In vitro* therapeutic efficacy evaluation showed that the PTT effect mediated by Fe_3_O_4_ nanoparticle-loaded hydrogels could obviously kill CT26 cancer cells, which was similar to that of Fe_3_O_4_ nanoparticles. Although Fe_3_O_4_ nanoparticles have been used for cancer PTT, we for the first time report the uses of Fe_3_O_4_ nanoparticle-loaded hydrogels for effective PTT. In view of the different characteristics of Fe_3_O_4_ nanoparticles, such as imaging, magnetism, and Fenton reaction, this platform may also be used for combinational therapy of cancer.

## Data Availability

The original contributions presented in the study are included in the article/supplementary material, further inquiries can be directed to the corresponding author.
